# A CAF-Fueled TIMP-1/CD63/ITGB1/STAT3 Feedback Loop Promotes Migration and Growth of Breast Cancer Cells

**DOI:** 10.3390/cancers14204983

**Published:** 2022-10-11

**Authors:** Angela Dittmer, Jürgen Dittmer

**Affiliations:** Clinic for Gynecology, University of Halle-Wittenberg, Ernst-Grube-Str. 40, 06120 Halle/Saale, Germany

**Keywords:** tissue inhibitor of metalloproteinase-1, carcinoma-associated fibroblasts, breast cancer, anti-estrogen

## Abstract

**Simple Summary:**

Carcinoma-associated fibroblasts (CAFs) are a major cellular component of the tumor microenvironment and influence cancer cell behavior in numerous ways. A large part of their actions is based on their high secretory activity, leading to the exposure of cancer cells to all kinds of bioactive factors, such as interleukin-6 (IL-6). Here, we present data showing that CAF-derived TIMP-1 activates STAT3 in breast cancer cells in cooperation with CD63 and integrin β1. In turn, STAT3 increases TIMP-1 secretion by breast cancer cells, leading to a TIMP-1/CD63/integrin β1/STAT3 positive feedback loop, which can be further fueled by IL-6. Functionally, this feedback loop is important for the CAF-induced increase in migratory activity and for CAF-induced resistance to the anti-estrogen fulvestrant.

**Abstract:**

TIMP-1 is one of the many factors that CAFs have been shown to secret. TIMP-1 can act in a tumor-supportive or tumor-suppressive manner. The purpose of this study was to elucidate the role of CAF-secreted TIMP-1 for the effects of CAFs on breast cancer cell behavior. Breast cancer cells were exposed to conditioned medium collected from TIMP-1-secreting CAFs (CAF-CM), and the specific effects of TIMP-1 on protein expression, migration and growth were examined using TIMP-1-specifc siRNA (siTIMP1), recombinant TIMP-1 protein (rhTIMP-1) and TIMP-1 level-rising phorbol ester. We observed that TIMP-1 increased the expression of its binding partner CD63 and induced STAT3 and ERK1/2 activation by cooperating with CD63 and integrin β1. Since TIMP-1 expression was found to be dependent on STAT3, TIMP-1 activated its own expression, resulting in a TIMP-1/CD63/integrin β1/STAT3 feedback loop. IL-6, a classical STAT3 activator, further fueled this loop. Knock-down of each component of the feedback loop prevented the CAF-induced increase in migratory activity and inhibited cellular growth in adherent cultures in the presence and absence of the anti-estrogen fulvestrant. These data show that TIMP-1/CD63/integrin β1/STAT3 plays a role in the effects of CAFs on breast cancer cell behavior.

## 1. Introduction

Breast cancer (BC) is the most frequent cancer among women and the leading cause of cancer-related death in women worldwide [1]. BC is a heterogenous disease, which is immunohistochemically subtyped in estrogen receptor α (ERα)-positive, human epidermal growth factor receptor 2 (Her2)-positive and triple-negative BCs [2,3]. ERα-positive BCs are treated with ERα-targeting drugs, such as tamoxifen and fulvestrant (FULV), and aromatase inhibitors [4,5,6]. Resistance to ERα-targeting drugs (endocrine resistance) is a common complication in treatment [7,8]. Endocrine resistance can be induced by numerous mechanisms, including the activations of the PI3K/AKT/mTOR and the Ras/Raf/MEK/ERK1/2 pathways [9,10].

The tumor microenvironment (TME) is an important component of tumors, playing a critical role in tumor progression and drug resistance [11]. Carcinoma-associated fibroblasts (CAFs) are among the different cell types being found in TME [12,13]. These activated fibroblasts contribute to tumor progression and drug resistance, including endocrine resistance [14]. An important part of the effects of CAFs on BC cells is the exposure of BC cells to a plethora of factors that CAFs secret. One prominent secreted factor is interleukin-6 (IL-6), which is responsible for a number of CAF effects on BCs [15]. This includes the activation of signal transducer and activator of transcription 3 (STAT3) and the upregulation of integrin β1 (ITGB1) expression. ITGB1 is an important surface receptor for the interaction of cells with the extracellular matrix [16]. Its inhibition leads to apoptosis of BC cells [17]. It is involved in metastasis, and its expression is associated with poor survival of BC patients [18,19]. Furthermore, ITGB1 expression was found to be permanently increased when BC cells are exposed to CAF-derived conditioned medium (CAF-CM) in the presence of FULV [20]. In addition, ITGB1 has been reported to induce tamoxifen resistance by activating the Ras/Raf/MEK/ERK1/2 pathway [21].

It is now well-established that ITGB1 can form a complex with CD63 and with the tissue inhibitor of metalloproteinase-1 (TIMP-1), whereby TIMP-1 binds directly to CD63 [22]. Through this complex, numerous signaling cascades, including the PI3K/AKT/mTOR and the Ras/Raf/MEK/ERK1/2 pathways, can be activated [23]. CD63 belongs to the tetraspanin superfamily of plasma membrane proteins and functions as a network former of supramolecular complexes. TIMP-1 is a soluble, secreted protein, which initially was found to inhibit metalloproteinases [24]. Later, TIMP-1 was appreciated as a multi-functional protein, which, for instance, can act anti-apoptotically on BC cells by activating the PI3K/AKT pathway [25]. CAFs secrete TIMP-1 in high amounts [26]. Therefore, CAF-derived TIMP-1 may contribute to the CAF effects on BC cells.

Prompted by our observation that one of the major factors secreted by CAFs is TIMP-1 [15], the aim of the present study was to elucidate the role of the TIMP-1/CD63/ITGB1 complex in the CAF effects on BC cells. We found that not only does CAF-CM lead to the exposure of BC cells to higher levels of extracellular TIMP-1, but that it also stimulates the BC cell-dependent secretion of this protein. Furthermore, we found that the levels of all three components of the TIMP-1/CD63/ITGB1 complex are increased in response to CAF-CM and that, through this complex, CAF-CM causes STAT3 activation in BC cells. Functionally, this complex was important for cellular growth and CAF-CM-induced migration.

## 2. Materials and Methods

### 2.1. Cell Lines/Sublines and Reagents

All cell lines/sublines were maintained in RMPI 1640 supplemented with 10% fetal calf serum (FCS) (PAN Biotech) in the absence of antibiotics. The same batch of FCS was used for cell propagation and all experiments. All cell lines and sublines were authenticated by SNP analysis (Genolytic or Eurofins). The establishment of the MCF-7 subline AnD5 and the preparation of CAF-CM are described elsewhere [20]. CAF-CM, AnD5-CM, BT474-CM and T47D-CM were collected after cells had been cultured for three days. Fulvestrant (LKT Laboratories), U0126 (Cell Signaling) and PMA (Calbiochem) were added to cells at a final concentration of 1 µM, 10 µM and 200 ng/mL, respectively. Stock solutions of fulvestrant, U0126 and PMA were prepared using DMSO. Mock treatments were performed by adding the corresponding amounts of DMSO. For incubation of cells with the TIMP-1 protein, rhTIMP-1 was added at a final concentration of 1 µg/mL. For treatment with IL-6, a final concentration of either 50, 250 or 500 ng/mL of rhIL-6 was used. Tunicamycin (Merck Chemicals) was added to the cells at a final concentration of 5 μg/mL.

### 2.2. Western Blot Analysis

Extraction of plasma membrane-bound, nuclear and cytosolic proteins and Western blot analysis were performed as previously described [20]. Proteins were either separated on a 10% gel or on a gradient gel (NuPAGE, Life Technology, Darmstadt, Germany). For the analysis of supernatants, 30 µL was applied unless stated otherwise. The primary antibodies used were as follows: rabbit polyclonal antibodies: anti-P(S473)-AKT (1:2000, D9E, Cell Signaling Technology, Frankfurt, Germany), anti-CD63 (1:2000, Proteintech, Planegg-Martinsried, Germany), anti-STAT3 (1:1000, 79D7, Cell Signaling Technology), anti-P(Tyr705)-STAT3 (1:1000, D3A7, Cell Signaling Technology), anti-TIMP1 (1:2000, C2C3, GeneTex, Eching, Germany); rabbit monoclonal antibodies: anti-integrin β1 (1:2000, EPR1040Y, Abcam, Berlin, Germany); mouse monoclonal antibodies: anti-(pan)AKT (1:2000, 40D4, Cell Signaling Technology) and anti-CD44 (1:2000, Lab vision, HCAM Ab-4). The anti-CAIX antibody was kindly provided by S. Pastorekova. Secondary antibody conjugates (anti-rabbit/anti-mouse horse radish peroxidase, 1:2000) were from Cell Signaling Technology. As antibodies against housekeeping proteins are not reliable markers for protein loading [27,28], protein loading was examined by either staining proteins with Fast Green (MERCK) or with Coomassie Blue (Blue G, SERVA Electrophoresis). To confirm equal protein loading of supernatants, a Fast Green-stained band corresponding to a large protein of the fetal calf serum is shown. For each condition, Western blot analyses were preformed two to three times. Full images can be found under Supplementary Materials File S1.

### 2.3. RNA Interference

Cells were transfected by si (small interference) RNAs as described [29]. Briefly, following electroporation using a Bio-Rad GenePulserX-cell (250 V, 800 μF), cells were seeded on a 10 cm culture dish and incubated for three days. The cells were then either lysed for protein extraction or used for growth or migration assays. The sequence of siTIMP1 is as follows: (sense: 5′-ACU GCA GGA UGG ACU CUU G-3′, antisense: 5′-CAA GAG UCC AUC CUG CAG U-3′). The sequences of siITGB1, siSTAT3 and siCD63 (CD63-2) and the control siRNA siLuc are decribed elsewhere [20,30,31]. All siRNAs were purchased from Eurofins.

### 2.4. Growth Assays

Growth activity was examined by an ATP/luciferase-based assay (Vialight Plus kit, Lonza, Verviers, Belgium) as described [15]. Briefly, for measuring growth activity of adherent cells, 10^4^ siRNA-transfected cells were added to each well of a 24-well plate. For determining growth activity in 3D suspension cultures, the cells were incubated in ultra-low attachment 96-well microplates (Corning, Steinfurt, Germany) at a density of 5 × 10^3^ cells/well. Under both culturing conditions, cells were grown for four days before they were lysed and ATP-measured by the luciferase-based assay in a Sirius luminometer (Berthold). To study the effect of CAF-CM on growth, CAF-CM was added to the medium at a ratio of 1 to 5.

### 2.5. Migration Assays

Migration activity was examined by wound-healing assays as described previously [29]. Briefly, after introducing a gap in the monolayer formed by siRNA-transfected cells, gap closure in the presence or absence of 20% CAF-CM was monitored for two days. For quantitation, the gap area as visible at 100-fold magnification was measured using Zeiss Axio Vision R 4.5 software (Zeiss, Jena, Germany). For each condition, the average gap area of three separate wounds was determined.

### 2.6. Statistical Analyses

For parametric tests, one-way ANOVA combined with Bonferroni correction for post-hoc analysis was used. A *p*-value of <0.05 was considered to indicate a statistically significant difference.

## 3. Results

### 3.1. Exposure of Breast Cancer Cells to High Concentrations of Extracellular TIMP-1 Modulates the Levels of Plasma Membrane Proteins Linked to TIMP-1 Function

**Figure 1:**
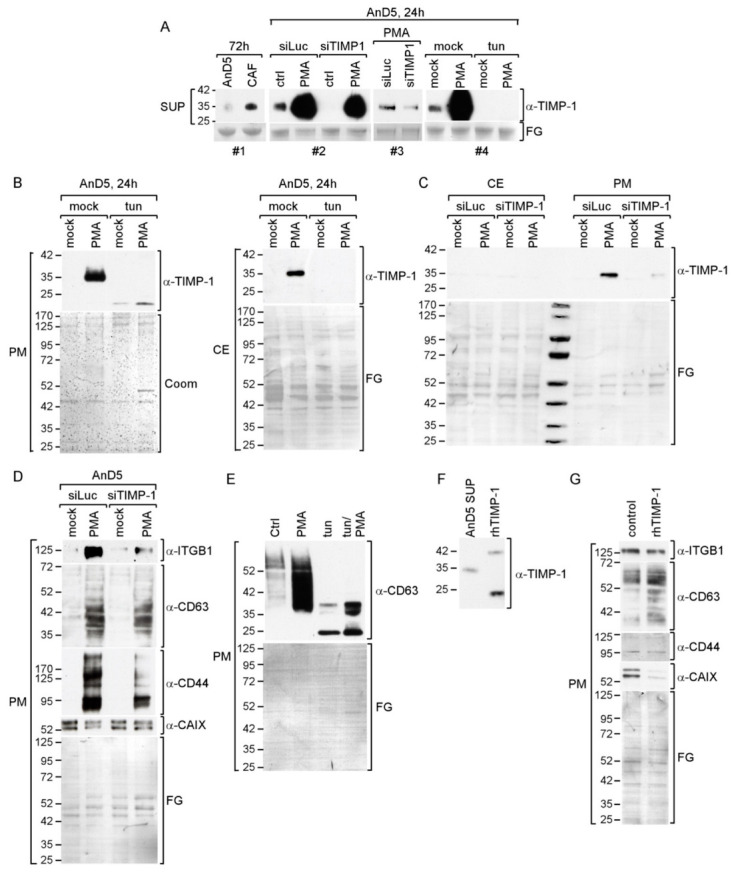
High extracellular TIMP-1 levels lead to a rise in CD63 expression in AnD5 cells. (**A**–**G**) Western blot analyses: (**A**) TIMP-1 levels in supernatants (sup) collected from AnD5 cells and CAFs after 72 h of culturing (panel #1), in AnD5 cells transfected either with control siRNA, siLuc or siTIMP1 in the presence or absence of PMA (panels #2, #3; note that, in panel #3, 5 µL instead of 30 µL sup was applied to prevent overloading of PMA-induced TIMP-1 protein) and after treatment of AnD5 cells with PMA and or tunicamycin (tun) (panel #4). (**B**,**C**) TIMP-1 abundances in the plasma membrane extract (PM) or cytosolic extract (CE) after culturing AnD5 cells in the presence or absence of PMA and/or tunicamycin (tun) (**B**) or in the presence or absence of PMA and siLuc or siTIMP1 (**C**). (**D**) Expression patterns of the CD63 protein in AnD5 cells treated with PMA and/or tunicamycin. (**E**) Expression of various membrane proteins as indicated in siLuc- or siTIMP-1-transfected AnD5 cells in the presence or absence of PMA. (**F**) Comparison of AnD5-secreted TIMP-1 with recombinant human TIMP-1 (rhTIMP-1) protein. (**G**) Effect of rhTIMP-1 on various membrane proteins in AnD5 cells as indicated. (**A**–**G**) To show equal protein loading, either the membrane was stained by Fast Green (FG) or the gel by Coomassie (Coom).

TIMP-1 is secreted by CAFs and cancer cells as well [26,32,33]. To compare TIMP-1 secretion by CAFs and BC cells, we chose 19TT, immortilized BC-derived CAFs, which, based on cytokine array data, we have previously shown to secret TIMP-1 [15], and AnD5 cells, an MCF-7 subline with a highly homogenous cell population and high responsiveness to CAF-CM [20]. Western blot analysis of the supernatants (SUP) collected after 3 days of culturing CAFs and AnD5 cells revealed an anti-TIMP-1 reactive protein of an apparent molecular weight (MW) of ~35 kD, which was more abundant in the SUP derived from CAFs than that from AnD5 cells (Figure 1A, panel #1), and which could be eliminated by a TIMP-1-specific siRNA (siTIMP1), but not by a control siRNA (siluc) (Figure 1A, panels #2 and #3). Consistent with previous data showing that secreted TIMP-1 is N-glycosylated [34], the TIMP-1-specific band disappeared after treating AnD5 cells with tunicamycin, an inhibitor of N-glycosylation (Figure 1A panel #4). These results show that 19TT-CAFs secrete more TIMP-1 than MCF-7-derived AnD5 BC cells. Hence, in the presence of 19TT-CAF-CM, AnD5 cells are exposed to a higher extracellular concentration of TIMP-1.

To examine the consequences of higher extracellular TIMP-1 concentrations for AnD5 cells, we first performed experiments with PMA, which has previously been demonstrated to increase TIMP-1 secretion by colon cancer cells [35]. We found that PMA also increases TIMP-1 secretion by AnD5 cells (Figure 1A, panels #2 and 4). Produced at a high level in the presence of PMA, the TIMP-1 protein could now be detected in the plasma membrane protein extract (PM) and in the cytosolic extract (CE) (Figure 1B), whereby its abundance in PM was much higher than in CE (Figure 1C), consistent with the finding that TIMP-1 binds to membrane proteins, such as CD63 [22]. Treatment with siTIMP-1 and tunicamycin confirmed that the PMA-induced anti-TIMP-1 reactive protein in the supernatant and in the plasma membrane protein extract was TIMP-1 and that it was N-glycosylated (Figure 1A, panels #3 and #4, Figure 1B,C).

We next examined whether the accumulation of the TIMP-1 protein at the plasma membrane in the presence of PMA coincides with higher abundances of membrane-bound CD63 and ITGB1. Indeed, PMA also increased the levels of both of these proteins (Figure 1D,E), whereby siTIMP1 reduced the PMA-induced expression of ITGB1 and also seemed to decrease basal and PMA-induced CD63 expression (Figure 1D). The many anti-CD63-reactive proteins are CD63 isotypes of different degrees of N-glycosylation [31], as confirmed by treating cells with tunicamycin (Figure 1E).

We additionally tested the PMA and siTIMP1 effects on two other membrane proteins, CD44 and carbon anhydrase IX (CAIX). CD44 is another binding partner of TIMP-1 [36], and CAIX is a downstream target of TIMP-1 and CD63 [37]. PMA increased CD44 expression, whereas it instead decreased the expression of CAIX (Figure 1D). SiTIMP-1 counteracted the PMA effect on CD44 and CAIX expression.

To further analyze the role of TIMP-1 for the expression of these membrane proteins, we used recombinant human (rh)TIMP-1, which in spite of its unglycosylated status (Figure 1F) is functional in terms of its ability to bind to CD63 [22]. RhTIMP-1 upregulated CD63 expression and down-regulated CAIX expression, but it failed to modulate CD44 and ITGB1 (Figure 1G). These data confirmed the regulatory effect of TIMP-1 on CD63 and on CAIX expression.

Collectively, these data suggest that TIMP-1 positively regulates the expression of its binding partner CD63 and downmodulates that of CAIX in AnD5 cells. Additionally, TIMP-1 seems to play a role in PMA-induced expression of ITGB1 and CD44.

### 3.2. TIMP-1 Is Responsible for the CAF-CM-Induced Upregulation of ITGB1

**Figure 2:**
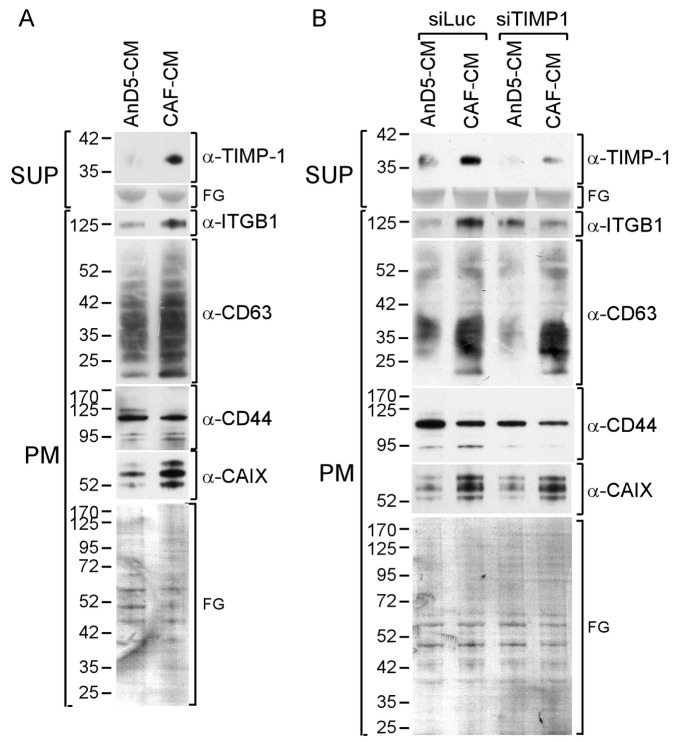
CAF-CM stimulates ITGB1 expression through TIMP-1. (**A**,**B**) Western blot analyses of plasma membrane extracts (PM) and supernantants (SUP) derived from AnD5 cells in the presence of CAF-CM or AnD5-CM without (**A**) or after transfection of AnD5 cells with the control siRNA siLuc or siTIMP1 (**B**). To show equal protein loading, the membranes were stained by Fast Green (FG).

We next examined the potential role of TIMP-1 in the expression of the four membrane proteins in the presence of CAF-CM. As shown before [20,30], CAF-CM was able to increase the levels of ITGB1 and CAIX (Figure 2A). The upregulation of the CAIX level in MCF-7 cells was previously found to be induced by the CAF-CM-induced rise in Bcl-3, but the reason for the upregulation of ITGB1 level was unclear [30]. We now show that siTIMP-1 prevents the CAF-CM-induced ITGB1 expression in AnD5 cells, suggesting that TIMP-1 is responsible for this effect (Figure 2B). Additionally, we found that CAF-CM raises the expression of CD63 (Figure 2A). However, this effect could not be reduced by siTIMP1, though the same siRNA lowered basal CD63 expression (Figure 2B). CD44 expression remained unchanged by siTIMP1. These data suggest that, in AnD5 cells, TIMP-1 is involved in the CAF-CM-dependent rise in ITGB1, but not in CAF-CM-induced upregulation of CD63 or CAIX expression.

### 3.3. CAF-CM Activates STAT3 and ERK1/2 by Stimulating the TIMP-1/CD63/ITGB1 Complex

**Figure 3:**
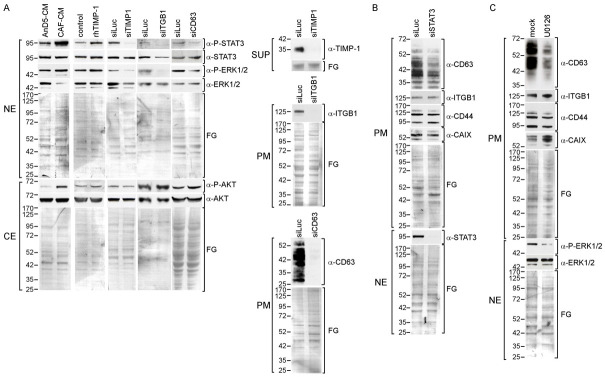
TIMP-1 activates STAT3 and ERK1/2 in concert with CD63 and ITGB1. (**A**–**C**) Western blot analyses of protein extracts from AnD5 cells. (**A**) The activities of STAT3, ERK1/2 and AKT proteins in AnD5 cells were determined by their phosphorylation statuses by using nuclear extracts (NE) or cytosolic extracts (CE) in the presence of AnD5-CM or CAF-CM, in the presence or absence of rhTIMP-1 or after transfection with siRNAs as indicated. The knock-down effects of siTIMP1, siITGB1 and siCD63 were confirmed by analyzing the supernatants (sup) or plasma membrane extracts (PM) for the abundances of TIMP-1, ITGB1 and CD63, respectively. To study the effects of AnD5-CM, CAF-CM and siRNAs, cells were incubated for three days, and cells were exposed to rhTIMP-1 for one day. (**B**,**C**) Effect of siSTAT3 and U0126 on the abundances of several membrane proteins as indicated. Protein extractions were performed after a three-day-treatment with control siRNA siLuc or siSTAT3 (**B**) or after a one-day-exposure to U0126 or mock (**C**). The effect of siSTAT3 on STAT3 expression (**B**) and of U0126 on ERK1/2 phosphorylation (**C**) was determined by analyzing nuclear extracts. To show equal protein loading, the membranes were stained by Fast Green (FG).

The TIMP-1/CD63/ITGB1 complex is able to activate a number of down-stream effectors, including ERK1/2 and AKT [23]. Furthermore, we and others have shown that ITGB1 or CD63 can upregulate STAT3 activity [20,38,39]. The STAT3 activity is commonly upregulated in BC lines in response to CAF-CM [15]. Additionally, some BC lines also activate ERK1/2 and/or AKT in response to CAF-CM. Since CAF-CM-treated AnD5 cells upregulate all of the three effectors [20] (Figure 3A), we wanted to know which of these

CAF-CM-induced effector activities were dependent on the TIMP-1/CD63/ITGB1 complex in these cells. We found that rhTIMP-1 mimicked the CAF-CM effects on STAT3 and ERK1/2 phosphorylation, whereas siTIMP1 downregulated STAT3 and ERK1/2 activities. Neither agent had an effect on AKT activity. An siRNA targeting CD63 (siCD63) and an siRNA directed against ITGB1 (siITGB1) showed similar effects as siTIMP1, suggesting that, in AnD5 cells, TIMP-1 regulates STAT3 and ERK1/2 activities by forming a complex with CD63 and ITGB1.

We next examined the importance of STAT3 and ERK1/2 for TIMP-1-regulated CD63 and CAIX expression by using an STAT3-specific siRNA (siSTAT3) to inhibit STAT3 expression and U0126 to inhibit ERK1/2 phosphorylation. Expression of CD63 was reduced by either agent (Figure 3B,C), suggesting that TIMP-1 regulates CD63 through both downstream effectors. In contrast, CAIX expression was upregulated by U0126 but not by siSTAT3, indicating that TIMP-1 downregulates CAIX expression by activating ERK1/2. Neither CD44 nor ITGB1, whose basal expression was not affected by TIMP-1, responded to the two agents.

### 3.4. CAF-CM Stimulates AnD5 Cells to Secret More TIMP-1 by Activating STAT3

**Figure 4:**
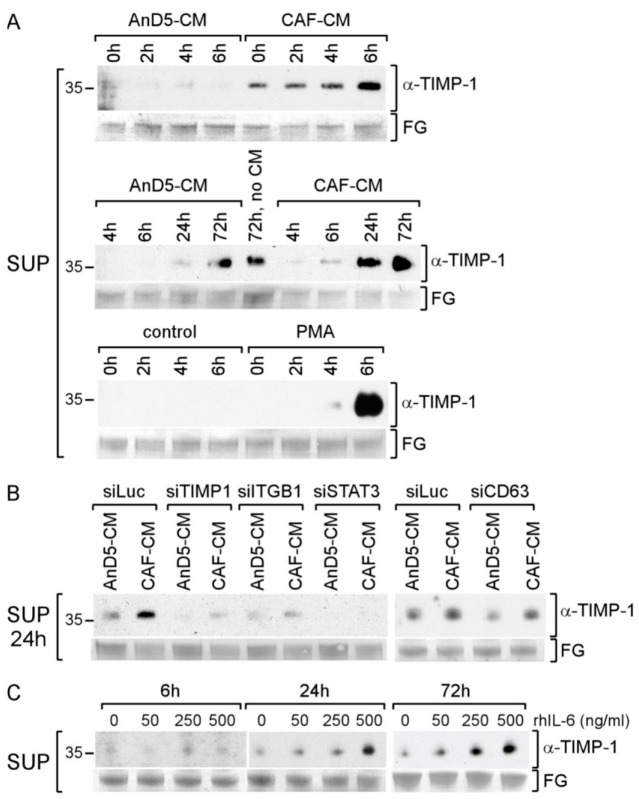
CAFs stimulate AnD5 cells to increase STAT3-dependent TIMP-1 secretion. (**A**–**C**) Western blot analyses of supernatants from AnD5 cells to determine the level of extracellular TIMP-1. (**A**) TIMP-1 levels after treatment of cells with AnD5-CM, CAF-CM (upper and middle panel), no CM (middle panel) or PMA or mock (lower panel) for the amount of time as indicated. (**B**) Effects of different siRNAs as indicated on TIMP-1 secretion. (**C**) Effects of various concentrations of rhIL-6 on TiMP-1 secretion. To show equal protein loading, the membranes were stained by Fast Green (FG).

As the TIMP-1 promoter contains a STAT3 recognition element through which TIMP-1 transcription can be activated [40], TIMP-1 expression in AnD5 cells may be upregulated by CAF-CM through STAT3. Indeed, CAF-CM stimulated the secretion of TIMP-1 by AnD5 cells, as indicated by a rise in the extracellular TIMP-1 level as early as 6 h after the start of the treatment (Figure 4A, upper panel). This can be compared to the 24 h required for a similar increase under control conditions (in the presence of AnD5-CM) (Figure 4A, middle panel). This effect was long-lasting, as even after 72 h, the level of TIMP-1 in the SUP of CAF-CM-treated cells was higher than in the SUP of cells treated with AnD5-CM or with no CM. However, the stimulatory effect of CAF-CM on TIMP-1 secretion was weaker than that of PMA, which already raised the TIMP-1 level after 4 h of incubation and induced a high accumulation of extracellular TIMP-1 after 6 h (Figure 4A, lower panel). These data indicate that CAF-CM treatment leads to higher exposure of AnD5 cells to extracellular TIMP-1, not only because CAFs secrete more TIMP-1 than AnD5 cells, but also because CAF-CM stimulates AnD5 cells to increase their TIMP-1 secretion.

To examine whether the CAF-CM-induced rise in TIMP-1 secretion by AnD5 cells depends on STAT3 activation, we treated cells with siSTAT3. We also transfected cells with siCD63 and siITGB1 to explore the possibility that TIMP-1 stimulates its own expression by forming a complex with CD63 and ITGB1. All three siRNAs reduced the extracellular concentration of TIMP-1 irrespective of whether CAF-CM was present or not (Figure 4B), suggesting that, in AnD5 cells, a positive TIMP-1/CD63/ITGB1/P-STAT3 feedback loop exists that keeps the P-STAT3 level high and through which CAF-CM can further activate STAT3.

IL-6 is a typical STAT3 activator [41], which is a major factor secreted by CAFs [15]. We therefore tested the ability of IL-6 to increase the extracellular level of TIMP-1 by using recombinant human (rh)IL-6. RhIL-6 increased the extracellular TIMP-1 level in a concentration-dependent manner (Figure 4C). Even after 6 h of treatment, a slight increase in the TIMP-1 level at higher IL-6 concentrations could be seen. At a lower concentration, a rise was observable after 24 h and 72 h. These data suggest that IL-6 is able to enhance extracellular TIMP-1 abundance and may therefore contribute to the maintenance of the TIMP-1/CD63/ITGB1/P-STAT3 feedback loop as fueled by CAF-CM. Hence, CAF-CM can increase TIMP-1-dependent STAT3 activity by exposing BC cells to a higher level of TIMP-1 and to IL-6.

### 3.5. Knockdown of Any Component of the TIMP-1/CD63/ITGB1/STAT3 Pathway Affects Growth and Migration of AnD5 Cells

STAT3 supports tumor progression by interfering with a number of cancer-promoting activities, such as proliferation, migration and anti-estrogen resistance [42,43]. If the concerted action of TIMP-1, CD63 and ITGB1 are a major way to activate STAT3 in AnD5 cells, downregulation of either component should have similar effects on STAT3-dependent cellular functions. We measured the effects of siTIMP1, siCD63, siITGB1 and siSTAT3 on migration as well as on cellular growth in the presence and absence of FULV and/or CAF-CM. CAF-CM has been shown to increase the migratory activity of AnD5 and MCF-7 cells and to desensitize these cells to FULV [15,20,30].

**Figure 5:**
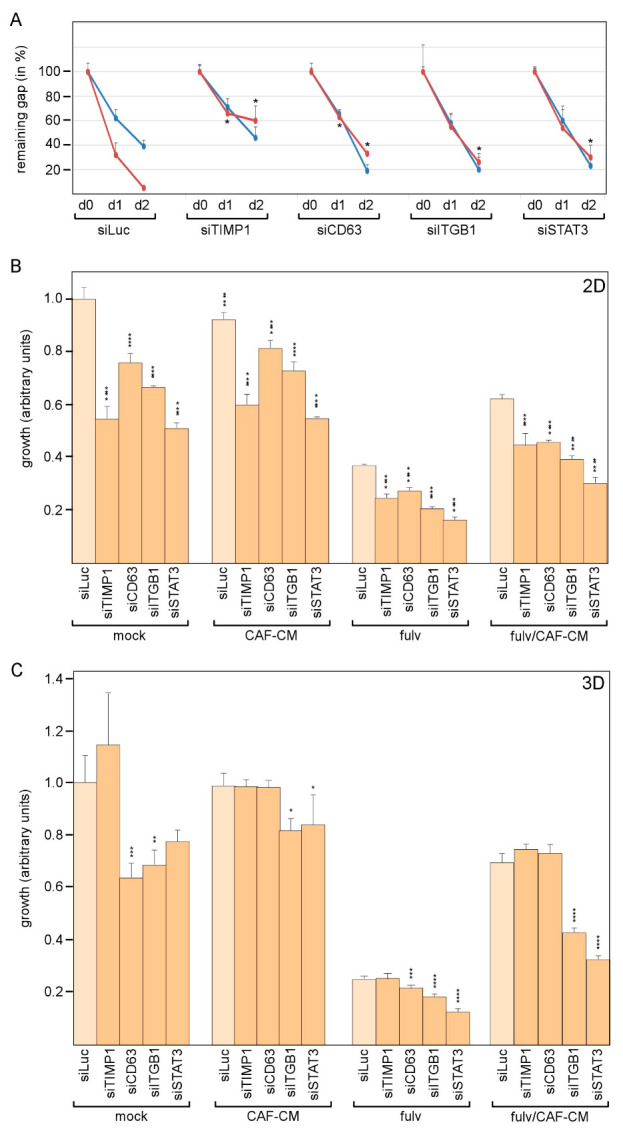
Knock-down of either component of the TIMP-1/CD63/ITGB1/STAT3 loop prevents CAF-CM-raised migration and affects cellular growth in 2D cultures. (**A**) Wound-healing assays of AnD5 cells transfected with an siRNA as indicated in the absence (blue line) or presence of CAF-CM (red line). (**B**,**C**) Growth assays in 2D adherent (**B**) and in 3D suspension cultures (**C**) in the presence of CAF-CM and/or FULV after AnD5 cells have been transfected with one of the five siRNAs as indicated. (**A**–**C**) Each bar represents the mean value ± S.D. (N ≥ 3). Statistical analyses were performed using Anova. Asterisks denote *p*-values: * *p* < 0.05, ** *p* < 0.01, *** *p* < 0.005, **** *p* < 0.001.

By performing wound healing assays to examine the migratory activity of AnD5 cells, we found that, as reported previously [20], CAF-CM accelerated gap closure under control conditions (siLuc) (Figure 5A). In the presence of siTIMP1, siCD63, siITGB1 or siSTAT3, the CAF-CM-induced increase in migratory activity was completely prevented, whereby, in the absence of CAF-CM, siCD63, siITGB1 and siSTAT3 promoted gap closure to some extent. Compared to cells transfected with the control siRNA siLuc, the differences in wound healing in the presence of CAF-CM were statistically significant for siTIMP-1- and siCD63-treated cells after one day and for cells transfected with any of the four siRNAs after two days. These data suggest that the TIMP-1/CD63/ITGB1/STAT3 feedback loop is responsible for the CAF-CM-induced rise in the migratory activity of AnD5 cells.

Next, the growth effects of the siRNAs in 2D adhesion and 3D suspension culture conditions were examined. As reported previously [15,30], CAF-CM desensitized cells to FULV, as indicated by increased growth of FULV-treated cells in the presence of CAF-CM, both in adhesion and suspension cultures (Figure 5B,C). In adhesion cultures, siTIMP1- siCD63-, siITGB1- and siSTAT3-transfected cells showed significantly lower growth activities than cells transfected with control siRNA siLuc under all conditions tested (Figure 5B). There were some differences in the strengths by which the four siRNAs affected growth, which ranged from 25 to 50% growth inhibition within four days of culturing. Overall, siSTAT3 had the strongest effect. The percent growth-inhibitory effects of the four siRNAs were the same in the presence and absence of CAF-CM, meaning that TIMP-1, CD63, ITGB1 and STAT3 were also important for CAF-CM-induced growth in the presence of FULV. These data show that all components of the TIMP-1/CD63/ITGB1/P-STAT3 feedback loop are required for AnD5 cellular growth in 2D adhesion cultures.

Further, in suspension cultures, siSTAT3 and siITGB1 inhibited growth by 20–50% and 20–40%, respectively, though they tended to be more effective in the presence of FULV (Figure 5C). In contrast, siTIMP1 did not affect growth under any condition, whereas siCD63 had some effect in the absence of CAF-CM. The data show that, in suspension cultures, AnD5 cellular growth is still dependent on ITGB1 and STAT3, particularly in the presence of FULV, but it does not require TIMP-1, nor is CD63 needed in the presence of CAF-CM. This suggests a disconnect of ITGB1-dependent P-STAT3 activation from the CD63/TIMP-1 complex in 3D suspension cultures, where cells are primarily engaged in cell–cell rather than in cell–ECM interactions.

### 3.6. CAF-CM Stimulates TIMP-1 Secretion also in Other BC Lines

**Figure 6:**
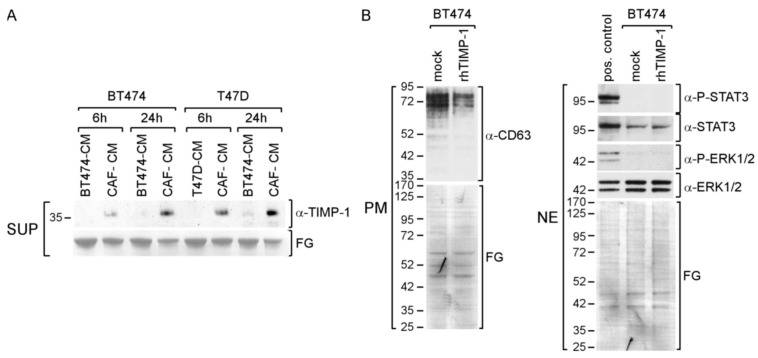
CAF-CM leads to an increase in exposure of BT474 and T47D cells to TIMP-1. (**A**) Western blot analysis of the supernatants (SUP) from BT474 and T47D cells after treatment with BT474-CM, T47D-CM or CAF-CM for the amounts of time as indicated. (**B**) Western blot analysis of plasma membrane extracts (PM) or nuclear extract (NE) from BT474 cells for the proteins and phospho-proteins as indicated. To show equal protein loading, the membranes were stained by Fast Green (FG).

Next, we examined the importance of TIMP-1 in BT474 and T47D BC lines. Both cell lines secrete TIMP-1, as indicated by a faint TIMP-1-specific band after 24 h of culturing in the presence of control CM, BT474-CM or T47D-CM, respectively (Figure 6A). In the presence of CAF-CM, the extracellular TIMP-1 abundance was much increased and further rose with time, suggesting that CAF-CM also stimulates these BC cells to secret more TIMP-1.

Given the more pronounced rise in TIMP-1 secretion by BT474 cells in the presence of CAF-CM, we chose this cell line for further analysis. Treatment with rhTIMP-1 failed to increase CD63, P-STAT3 and P-ERK1/2 levels (Figure 6B), suggesting that, in BT474 cells, TIMP-1 is not involved in activating STAT3 and ERK1/2 and, as a consequence, does not regulate CD63 expression. Hence, a CAF-CM-induced increase in the extracellular TIMP-1 level does not necessarily result in activating the CD63/ITGB1 complex to activate STAT3 or ERK1/2. Of note, as indicated by their higher apparent MWs, the CD63 isotypes in BT474 seem to be more highly glycosylated as compared to those of AnD5 cells (compare Figure 6B to Figure 1D,G).

## 4. Discussion

**Figure 7:**
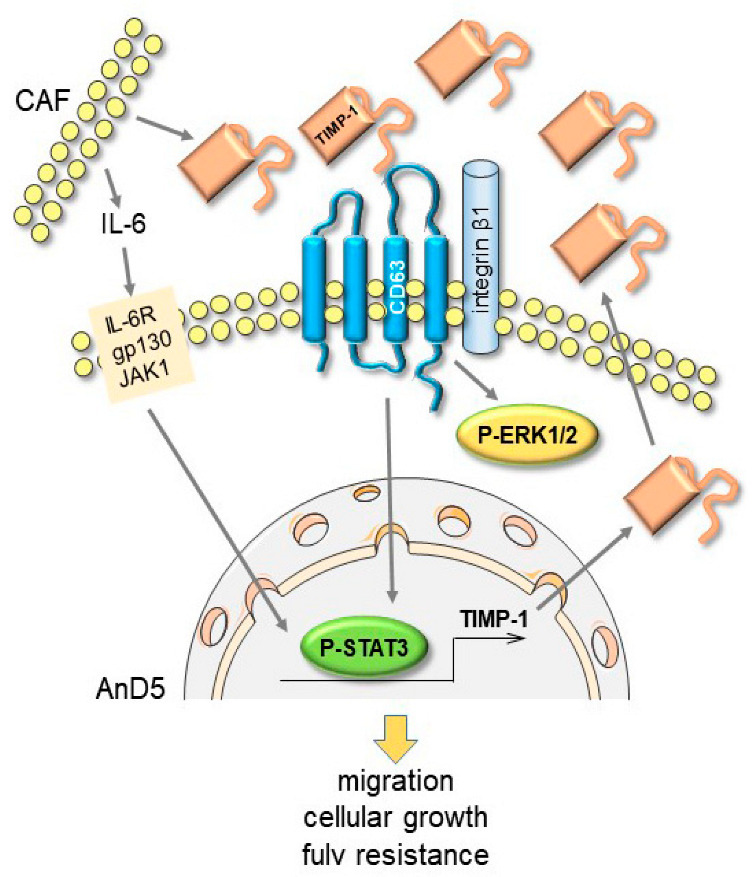
A schematic illustrating a TIMP-1/CD63/ITGB1/P-STAT3 positive feedback loop as suggested by the results presented above. Exposed to CAF-CM-derived TIMP-1, BC cells are stimulated to activate STAT3 and to produce more TIMP-1, which then leads to further STAT3 activation. In addition, IL-6 secreted by CAFs helps to maintain STAT3 activation, thereby fueling the feedback loop. IL-6R = IL-6 receptor, gp130 = glycoprotein 130, JAK1 = Janus kinase 1.

Activation of STAT3 is a common response of BC cells when they are exposed to the cocktail of factors secreted by CAFs [15]. A major component of the CAF-derived secretome is IL-6 [15], which, by stimulating the IL-6 receptor/gp130/JAK1/STAT3 cascade, is a key driver of STAT3 activation [41]. Cell lines that upregulate STAT3 activity in response to CAF-CM do the same upon treatment with rhIL-6 [15]. Here, we show an alternative way through which CAFs can activate STAT3 in BC cells. Our data suggest that STAT3 can be activated by a cooperative interaction of TIMP-1, CD63 and ITGB1. The data support a hypothesis by which CAF-secreted TIMP-1 initiates a positive feedback loop, which leads to a higher STAT3 activation level, which then increases TIMP-1 secretion (Figure 7).

The STAT3-responsiveness of TIMP-1 allows IL-6 to further fuel this loop. So far, typical downstream effectors of the TIMP-1/CD63/ITGB1 complex include ERK1/2 and AKT, but not STAT3 [23], though we and others have linked either CD63 or ITGB1 to STAT3 activity [20,38,39]. Hence, to our knowledge, our data show for the first time that the TIMP-1/CD63/ITGB1 complex can be a major regulator of STAT3 activity in BC cells. Obviously, this feedback loop is important for the CAF-CM effect on BC cells, as the CAF-CM-induced rise in migratory activity of AnD5 cells is blunted by knock-down of any component of this loop. 

If examining the effect of CAF-derived TIMP-1, the amount of TIMP-1 secreted by BC cells have to be considered. We found that, under the same conditions of culturing, CAFs secret much more TIMP-1 than MCF-7-derived AnD5, BT474 and T47D cells. This is in agreement with previously published data showing that LoVo colon cancer cells produce an order of a magnitude less TIMP-1 than CAFs [26]. Strikingly, CAF-CM treatment also increased TIMP-1 secretion by BC cells, an effect most prominent in AnD5 cells. Therefore, upon incubation with CAFs, BC cells are exposed to a much higher extracellular concentration of TIMP-1, whether directly derived from CAFs or indirectly by CAF-induced stimulation of BC cells’ own secretion.

To study the consequences of a higher extracellular concentration of TIMP-1 for the BC cells, we first focused on the expression of certain membrane proteins, which have been shown to be linked to TIMP-1. Including experiments with rhTIMP-1 and TIMP-1 secretion promoter PMA, we found CD63 to be a major target of TIMP-1. Consistent with this result, it was reported that in glioma specimens, TIMP-1 expression correlated with that of CD63 [44]. Inhibition of STAT3 and ERK1/2 activities resulted in a decline in CD63 expression, suggesting that both downstream effectors are involved in CD63 regulation by TIMP-1. The CD63 promoter contains an AP-1 binding site, which explains the PMA responsiveness of the CD63 gene [45]. In addition, the AP-1 site may be important for ERK1/2 to upregulate CD63 expression, as ERK1/2 has been shown to increase the abundance of the AP-1 component c-Fos [46].

We have previously reported that ITGB1 expression was upregulated by the treatment of MCF-7 and AnD5 cells with CAF-CM [20,30]. We now show that this effect is caused by a higher concentration of extracellular TIMP-1. We also show that TIMP-1 is involved in PMA-induced upregulation of ITGB1, but not in its basal expression.

The TIMP-1 effect on the expression of CAIX, a regulator of cellular pH [47], was of interest, since TIMP-1 in concert with CD63 was found to upregulate CAIX expression in triple-negative MCF10AneoT and MCF10CA1h BC cells, thereby inducing extracellular acidosis [37]. Here, we show that, contrary to these previous results, TIMP-1 instead downregulated CAIX plasma membrane abundance in ERα-positive AnD5 cells. It is possible that TIMP-1 differently regulates CAIX expression in ERα-positive and triple-negative BC cells. CAIX expression in AnD5 cells was highly upregulated by U0126 but not by STAT3 knock-down, suggesting that TIMP-1 downregulates CAIX expression in AnD5 cells by activating ERK1/2. A previous report has shown that the CAIX promoter is responsive to ERK1/2 [48]. However, in the same report, U0126 acted in an inhibitory manner on CAIX promoter activity and expression, suggesting that ERK1/2 can also promote CAIX expression. Clearly, the rise in CAIX expression in CAF-CM-treated cells is not linked to TIMP-1. We have previously shown by using MCF-7 cells that CAF-CM upregulates CAIX expression through a CAF-CM-induced increase in the expression of Bcl-3 [30], a IκB-like protein involved in anti-estrogen resistance [49]. CAIX has also been shown to play a role in drug resistance [47].

TIMP-1 has been reported to act in a growth-stimulatory manner on many cancer cells, including BC cells [50,51,52], and CD63 and ITGB1 were linked to drug resistance of BC cells [21,31]. Here, we show that the TIMP-1/CD63/ITGB1/STAT3 feedback loop is of functional importance for AnD5 cells, as knock-down of any of these factors significantly inhibited cellular growth in 2D adherent cultures and reduced the effect of CAF-CM on FULV resistance. However, in 3D suspension cultures, where these cells form spheroids, TIMP-1 and, in the presence of CAF-CM, also CD63 were dispensable for growth, though ITGB1 and STAT3 were still important. Alternative interaction partners have been reported to be involved in ITGB1-dependent STAT3 activation. Among them are gp130, a downstream effector of IL-6 [39]. Interestingly, in the presence of FULV, rhIL-6 promotes growth in 3D, but not 2D cultures of MCF-7 and AnD5 cells [15]. It is therefore tempting to speculate that, by transferring cells from 2D to 3D cultures, ITGB1 switches from CD63 to gp130 as an interaction partner to activate STAT3.

Other than the difference in the requirement of TIMP-1 and CD63 for STAT3 activity in 2D and 3D cultures, the TIMP-1/CD63/ITGB1/STAT3 feedback loop may exist only in certain BC cells. We found this loop in the MCF-7-derived cell line AnD5 with a high responsiveness to CAF-CM by showing higher expression of a number of proteins and activated proteins, enhanced growth in the presence of FULV and highly increased migration [20]. In contrast, in BT474 cells, which do not show higher expression of ITGB1 and CD63 in response to CAF-CM and whose growth activity in the presence of FULV is only weakly affected by CAF-CM [20], no stimulatory effect of TIMP-1 on STAT3 activity could be detected. Interestingly, in BT474 cells, CD63 seems to be more heavily glycosylated than in AnD5 cells. The extent of N-glycosylation has been shown to interfere with CD63 function [31]. Particularly, higher N-glycosylation of CD63 was linked to higher abundance of the MDR1 protein at the plasma membrane. It is possible that the degree of CD63 glycosylation also interferes with CD63′s ability to interact with TIMP-1.

As mentioned above, CD63 is not the only plasma membrane protein that is able to interact with TIMP-1. CD44 and CD82 are two additional proteins, which have been reported to physically interact with TIMP-1, whereby the interaction of TIMP-1 with CD44 is bridged through proMMP-9 [36,53]. CD44 expression in AnD5 cells could be upregulated by PMA, which was at least partly depended upon TIMP-1, but, unlike CD63 expression, was not induced by CAF-CM. Nor was CD44 expression affected by STAT3 knock-down, which suggests that CD44 is not involved in the TIMP-1/CD63/ITGB1/STAT3 feedback loop. Like CD63, CD82 belongs to the tetraspanin superfamily [54] and is able to interact with ITGB1 [55]. Whereas CD63 binds to the C-terminal domain of TIMP-1 [22], CD82 binds to its N-terminal region [53]. CD82 has been found to be a metastatic suppressor and shown to suppress migration and/or invasion of cancer cells [54]. Further, BC cells showed reduced migratory activities in the presence of CD82 [56]. Zhang and co-workers demonstrated that CD82 mediated the suppressive effect of TIMP-1 on the migratory activity of the pancreatic cancer PANC-1 cells [53]. Since TIMP-1 showed a migration-promoting effect on AnD5 cells, a contribution of CD82 on this effect is rather unlikely.

In our experiments, the BC-derived CAF isolate we were using secrets both TIMP-1 and IL-6 [15]. This may not be a feature of all CAFs in BC, since CAFs are a heterogenous group of cells, whose features also depend on the cancer type to which they are associated [57]. For instance, in pancreatic cancer, two major types of CAFs could be distinguished, myofibroblast-like CAFs (myCAFs) and inflammatory CAFs (iCAFs), the latter being characterized by strong expression of IL-6 [58]. In human BCs, four different subtypes of CAFs have been identified, which are not distinguished by their abilities to secret IL-6 but rather by the expression of other proteins, including the cytokines SDF1 and CCL11 [59]. In murine BCs, six CAF subtypes were reported, of which one subtype showed high expression of TIMP-1 [60]. TIMP-1-expressing CAFs are also found in other cancers. For instance, in colon cancer, TIMP-1-expressing CAFs are frequently present in those cancers associated with Crohn’s disease [61], a chronic inflammatory, IL-6-dependent disease [62]. Hence, the establishment of a TIMP-1/IL-6 loop to increase STAT3 activity may depend on cancer type and CAF subtype.

Other cellular components of the TME may also contribute to the extracellular TIMP-1 level in the cancerous lesion. For instance, tumor-associated macrophages (TAMs) have been found to express TIMP-1 depending on their phenotype. M1-like, but not M2-like TAMs were shown to secret TIMP-1 and IL-6 [63,64]. Although M1-like TAMs show generally anti-tumoral effects, they may induce chronic inflammation [65], whereas the TIMP-1/IL-6 loop might foster STAT3 activation in cancer cells.

## 5. Conclusions

STAT3 is a well-known factor involved in tumor progression. It affects proliferation, migration and survival and is often a target for stromal cells to modulate cancer behavior. A common STAT3 activator that is secreted in substantial amounts by CAF is IL-6. Now, we have shown that, in addition, CAFs may fuel a TIMP-1/CD63/ITGB1/STAT3 feedback loop to increase the STAT3 activity level in BC cells. A higher activity of this feedback loop is caused by CAFs by exposing BC cells to high levels of TIMP-1. Additionally, IL-6 may support this loop by further activating STAT3 through the gp130/JAK pathway. The feedback loop has the potential to mediate the CAF-induced increase in migration and to promote cellular growth. It seems, however, that this loop is not generally used by BC cells to maintain high STAT3 activity. Differences in BC cells to respond to TIMP-1 are just another example of how differently environmental cues are perceived by different cells.

## Data Availability

The data presented in this study are available in this article.

## Supplementary Materials

The following supporting information can be downloaded at: https://www.mdpi.com/article/10.3390/cancers14204983/s1. File S1: Original blots.
