# Supplementary material for: A CAF-Fueled TIMP-1/CD63/ITGB1/STAT3 Feedback Loop Promotes Migration and Growth of Breast Cancer Cells
Source: Cancers (Basel). 2022 Oct 11;14(20):4983. doi: 10.3390/cancers14204983 (PMC9599197; doi:10.3390/cancers14204983)

Fig. 1A, panel #1
α-TIMP-1

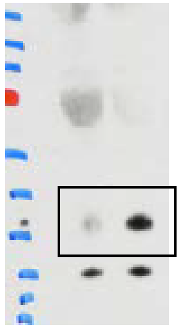

Fig. 1A
panel #2
α-TIMP-1

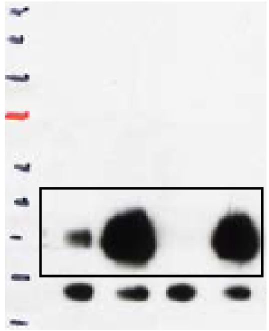

Fig. 1A
panel #3
α-TIMP-1

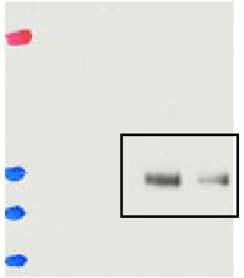

Fig. 1A, panel #4
α-TIMP-1

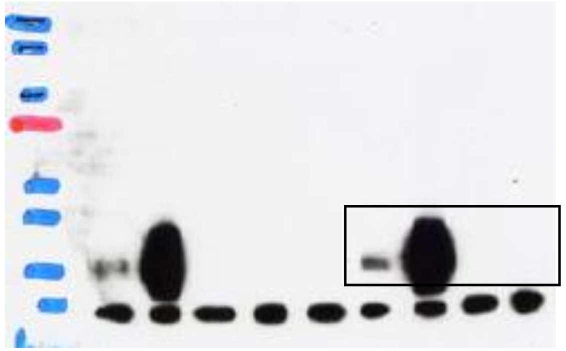

Fig. 1A, panel #1
FastGreen

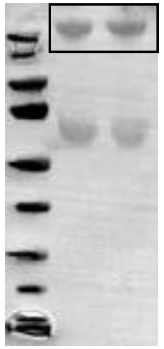

Fig. 1A
panel #2
FastGreen

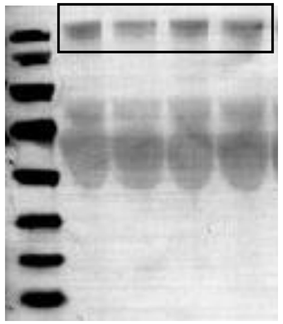

Fig. 1A
panel #3
Fastgreen

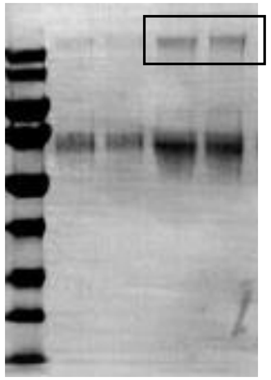

Fig. 1A, panel #4
FastGreen

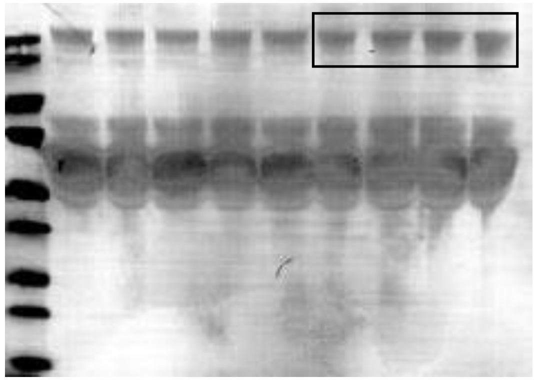

Fig. 1B, PM, α-TIMP-1

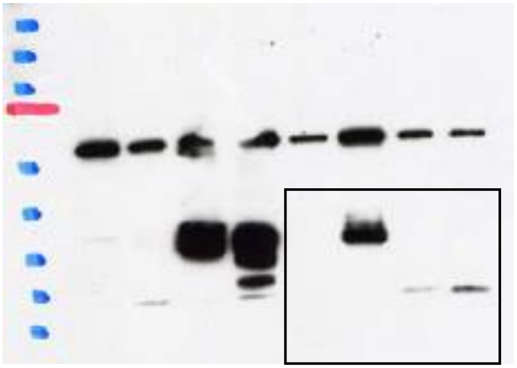

Fig. 1B, CE, α-TIMP-1

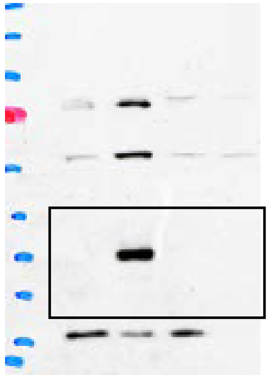

Fig. 1D, α-ITGB1

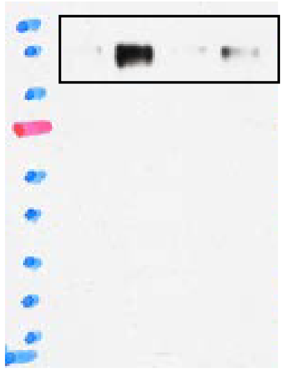

Fig. 1D, α-CAIX

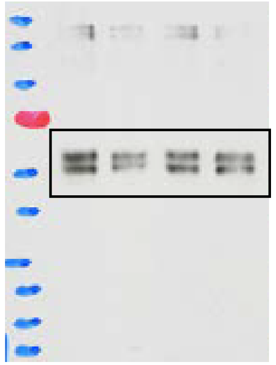

Fig. 1E, α-CD63

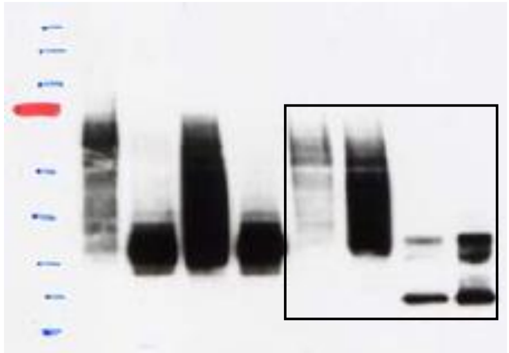

Fig. 1B, PM, Coomassie

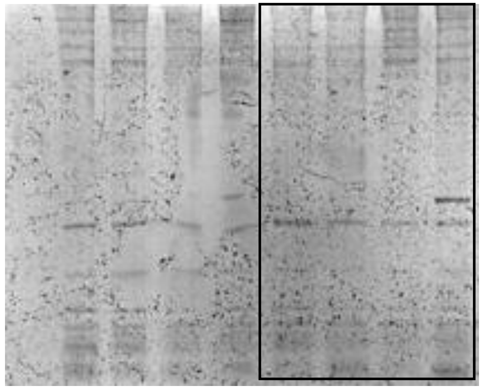

Fig. 1B, CE, FastGreen

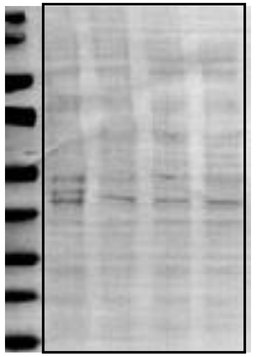

Fig. 1D, α-CD63

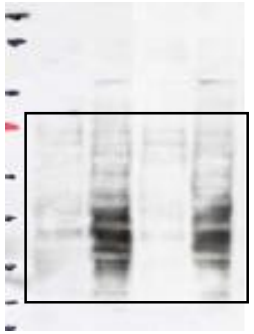

Fig. 1D, FastGreen

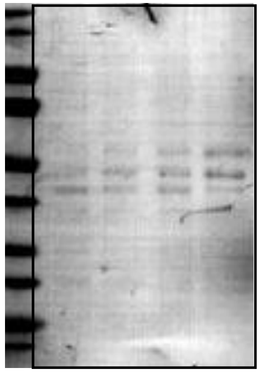

Fig. 1E, FastGreen

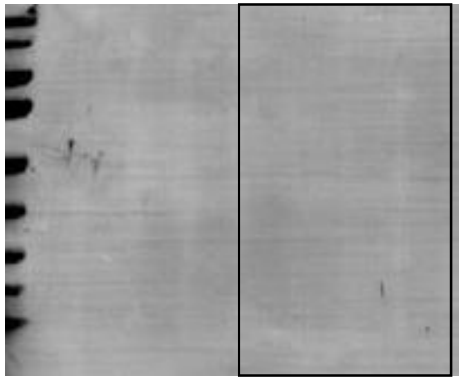

Fig. 1D, α-CD44

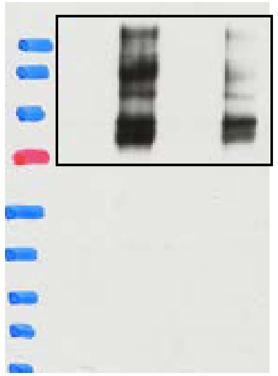

Fig. 1F

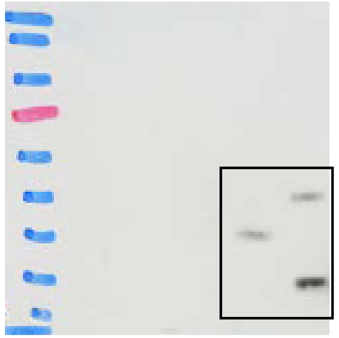

Fig. 1C, α-TIMP-1

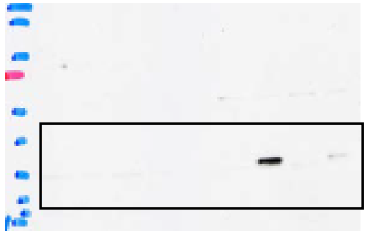

Fig. 1C, FastGreen

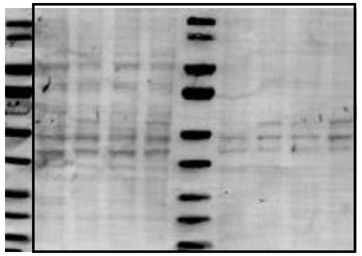

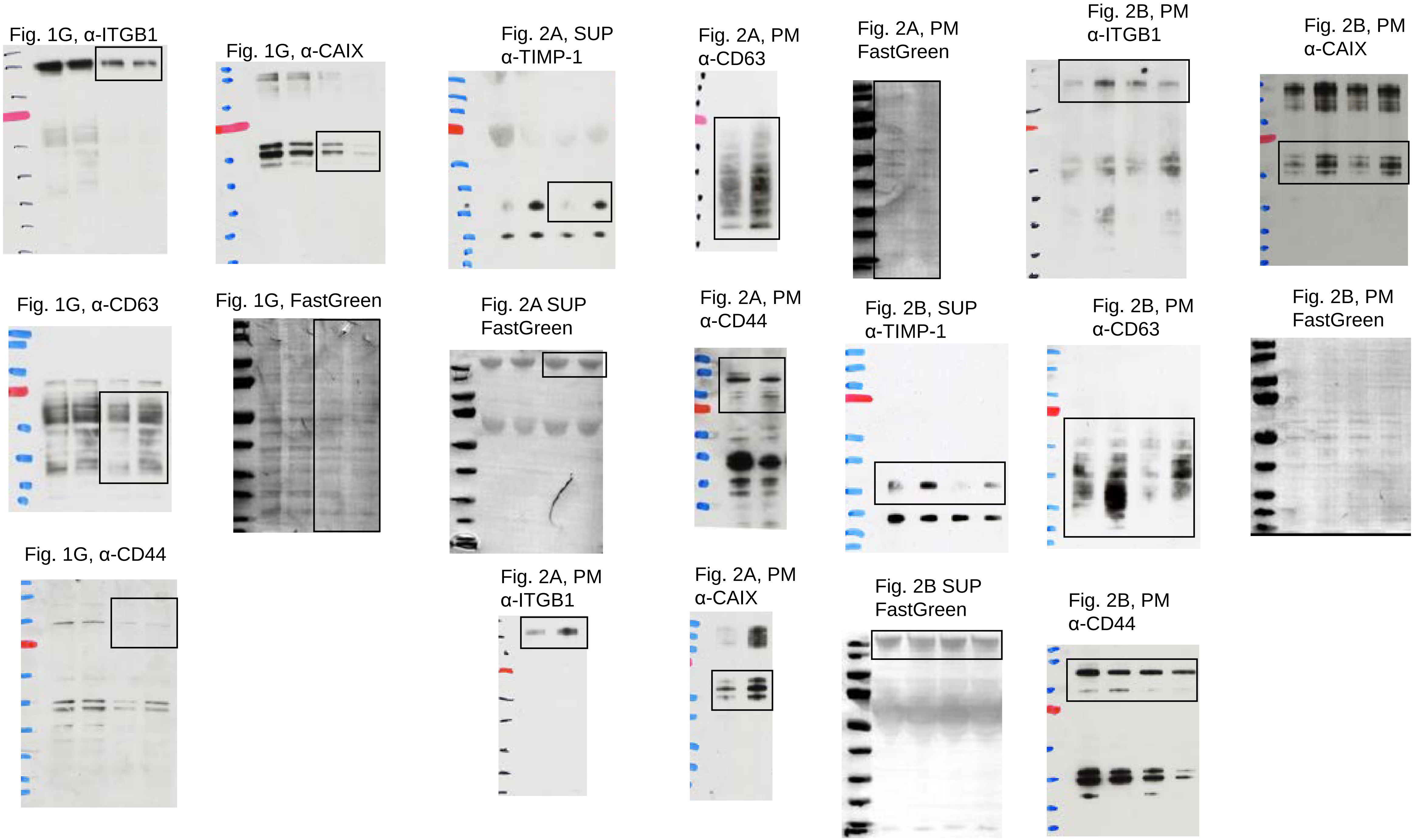
Fig. 1G, α-ITGB1
Fig. 1G, α-CAIX
Fig. 2A, SUP
α-TIMP-1
Fig. 2A, PM
α-CD63
Fig. 2A, PM
FastGreen
Fig. 2B, PM
α-ITGB1
Fig. 2B, PM
α-CAIX
Fig. 1G, α-CD63
Fig. 1G, FastGreen
Fig. 2A SUP
FastGreen
Fig. 2A, PM
α-CD44
Fig. 2B, SUP
α-TIMP-1
Fig. 2B, PM
α-CD63
Fig. 2B, PM
FastGreen
Fig. 1G, α-CD44
Fig. 2A, PM
α-ITGB1
Fig. 2A, PM
α-CAIX
Fig. 2B SUP
FastGreen
Fig. 2B, PM
α-CD44

Fig. 3A, α-P-STAT3
± CAF-CM

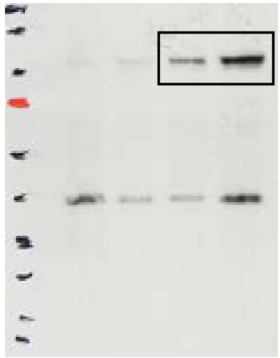

Fig. 3A, α-STAT3
± CAF-CM

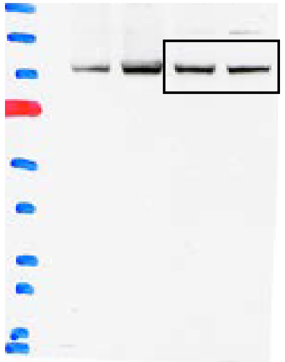

Fig. 3A, α-P-AKT
± CAF-CM

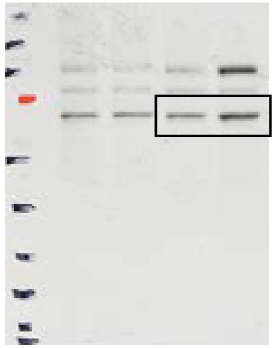

Fig. 3A, α-P-ERK1/2
± recTIMP-1

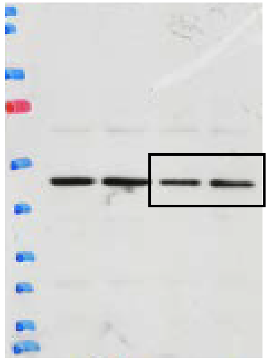

Fig. 3A, α-ERK1/2
± recTIMP-1

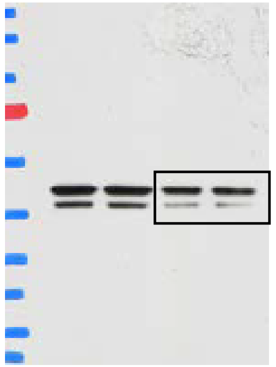

Fig. 3A, α-P-AKT
± recTIMP-1

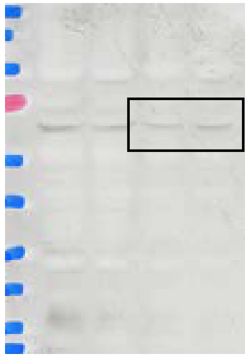

Fig. 3A, α-P-ERK1/2
± CAF-CM

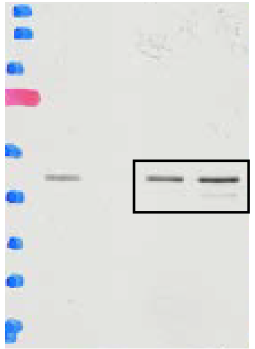

Fig. 3A, α-ERK1/2
± CAF-CM

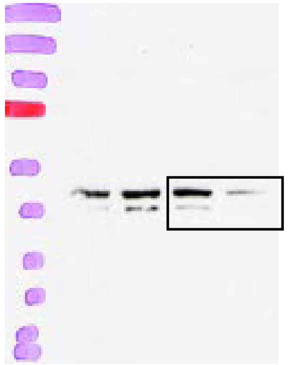

Fig. 3A, α-AKT
± CAF-CM

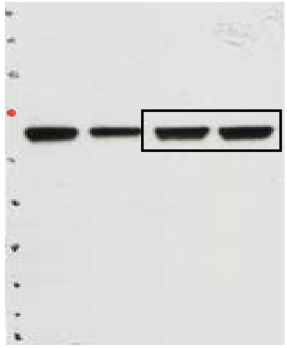

Fig. 3A, α-P-STAT3
± recTIMP-1

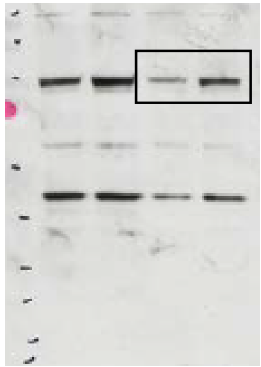

Fig. 3A, α-STAT3
± recTIMP-1

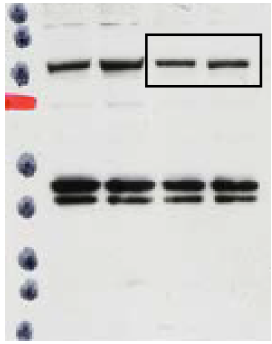

Fig. 3A, α-AKT
± recTIMP-1

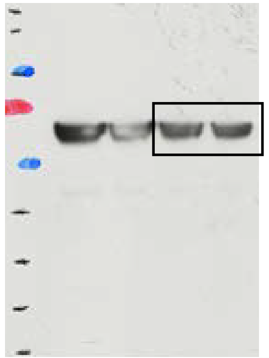

Fig. 3A, FASTGREEN
± CAF-CM, NE

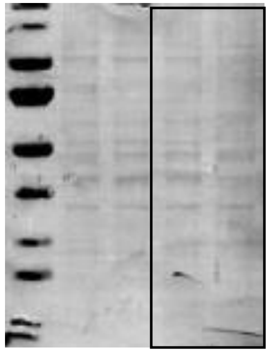

Fig. 3A, FASTGREEN
± CAF-CM, CE

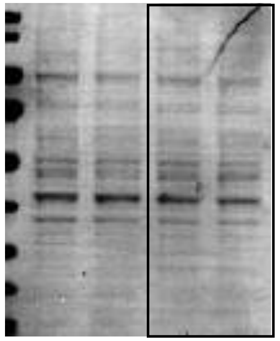

Fig. 3A, FastGreen
± recTIMP-1, NE

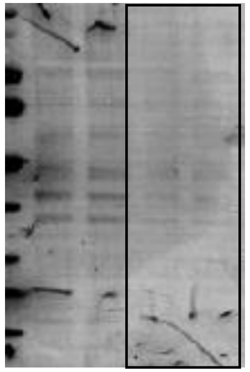

Fig. 3A, FastGreen
± recTIMP-1, CE

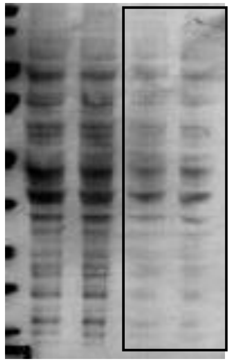

Fig. 3A, α-P-STAT3
siTIMP1 vs. siLuc

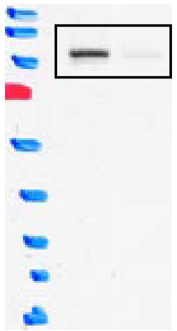

Fig. 3A, α-STAT3
siTIMP1 vs. siLuc

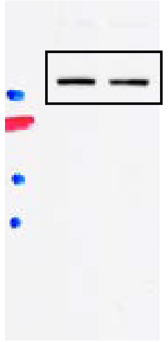

Fig. 3A, α-P-AKT
siTIMP1 vs. siLuc

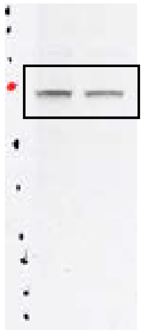

Fig. 3A, α-P-STAT3
siITGB1 vs. siLuc

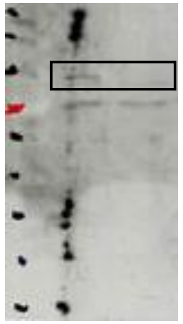

Fig. 3A, α-STAT3
siITGB1 vs. siLuc

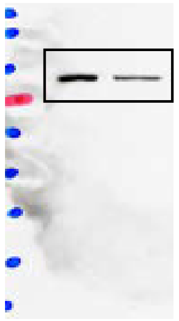

Fig. 3A, α-P-AKT
siITGB1 vs. siLuc

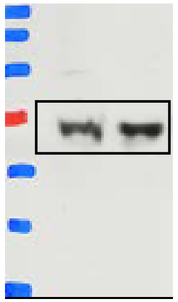

Fig. 3A, α-ITGB1
siITGB1 vs. siLuc

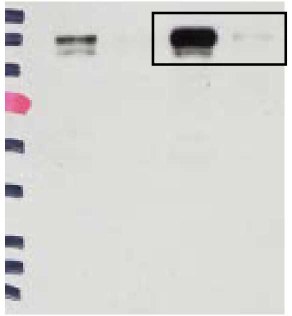

Fig. 3A, α-P-ERK1,2
siTIMP1 vs. siLuc

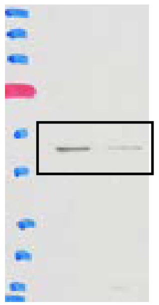

Fig. 3A, α-ERK1,2
siTIMP1 vs. siLuc

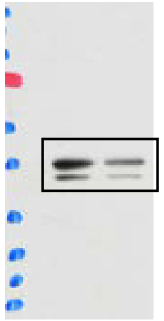

Fig. 3A, α-AKT
siTIMP1 vs. siLuc

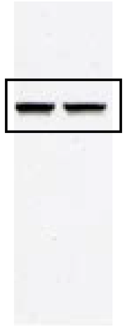

Fig. 3A, α-P-ERK1/2
siITGB1 vs. siLuc

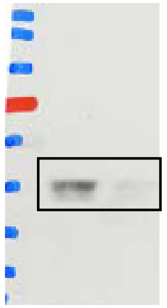

Fig. 3A, α-ERK1/2
siITGB1 vs. siLuc

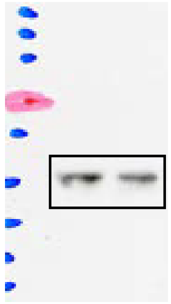

Fig. 3A, α-AKT
siITGB1 vs. siLuc

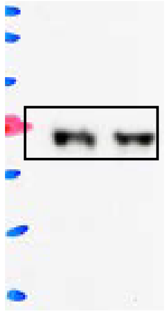

Fig. 3A, Coomassie
siITGB1 vs. siLuc, PM

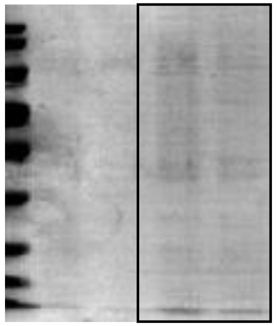

Fig. 3A, FastGreen
siTIMP1 vs. siLuc

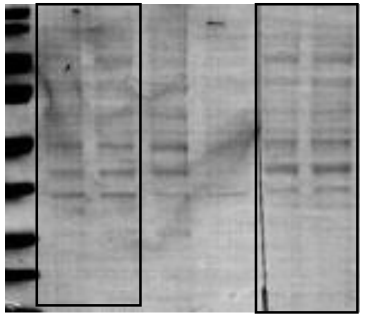

Fig. 3A, FastGreen
siITGB1 vs. siLuc, NE

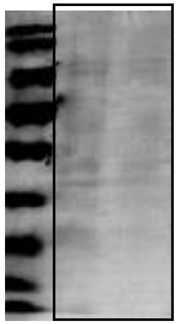

Fig. 3A, FastGreen
siITGB1 vs. siLuc, CE

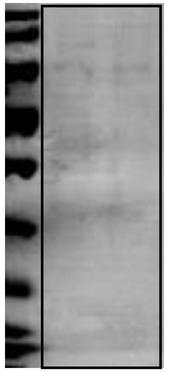

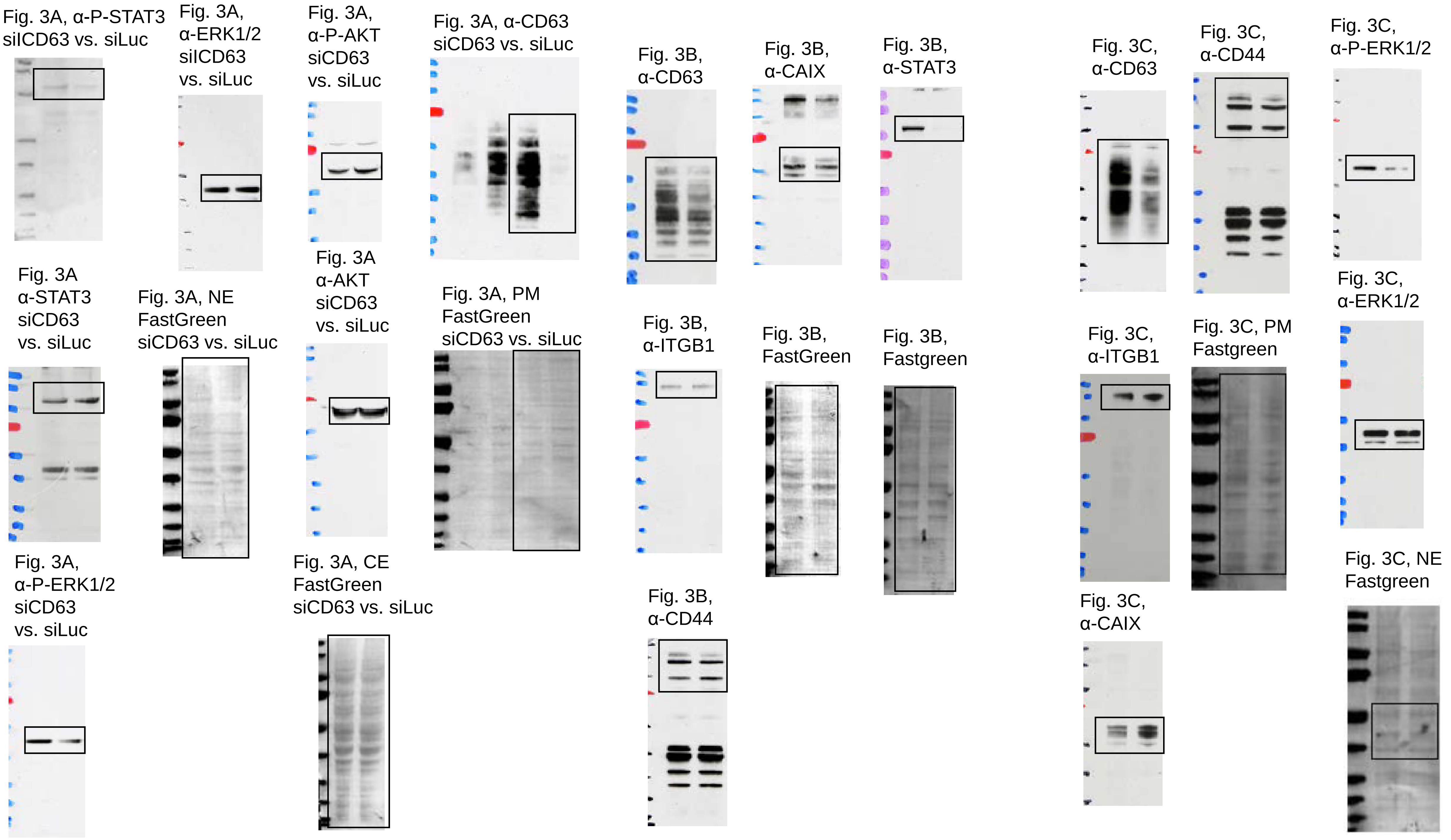
Fig. 3A, α-P-STAT3 siICD63 vs. siLuc
Fig. 3A, α-ERK1/2 siICD63 vs. siLuc
Fig. 3A, α-P-AKT siCD63 vs. siLuc
Fig. 3A, α-CD63 siCD63 vs. siLuc
Fig. 3A α-STAT3 siCD63 vs. siLuc
Fig. 3A, NE FastGreen siCD63 vs. siLuc
Fig. 3A α-AKT siCD63 vs. siLuc
Fig. 3A, PM FastGreen siCD63 vs. siLuc
Fig. 3A, α-P-ERK1/2 siCD63 vs. siLuc
Fig. 3A, CE FastGreen siCD63 vs. siLuc
Fig. 3B, α-CD63
Fig. 3B, α-CAIX
Fig. 3B, α-STAT3
Fig. 3B, α-ITGB1
Fig. 3B, FastGreen
Fig. 3B, Fastgreen
Fig. 3B, α-CD44
Fig. 3C, α-CD63
Fig. 3C, α-CD44
Fig. 3C, α-P-ERK1/2
Fig. 3C, α-ERK1/2
Fig. 3C, α-ITGB1
Fig. 3C, PM Fastgreen
Fig. 3C, α-CAIX
Fig. 3C, NE Fastgreen

Fig. 4A, α-TIMP-1_upper panel

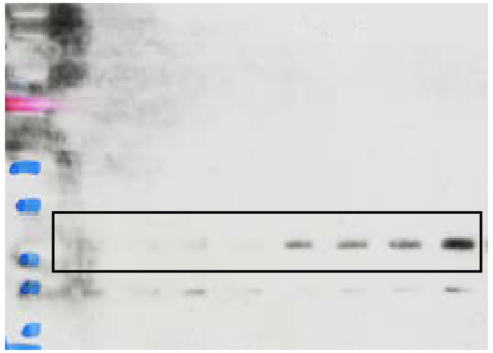

Fig. 4A, FastGreen_upper panel

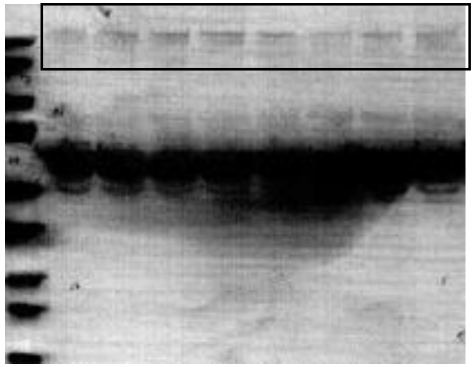

Fig. 4A, α-TIMP-1_middle panel

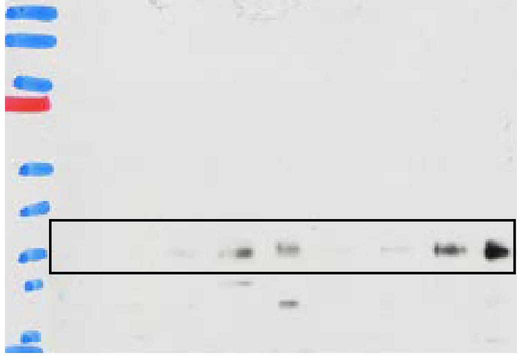

Fig. 4A, FastGreen_middle panel

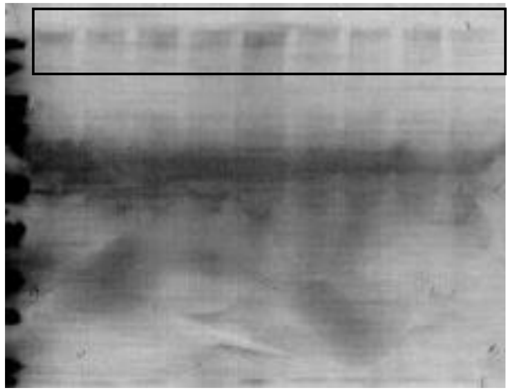

Fig. 4A, α-TIMP-1_lower panel

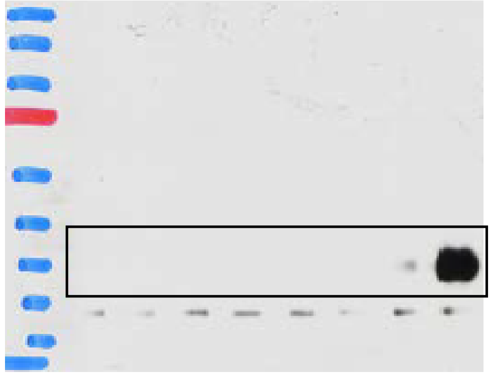

Fig. 4A, FastGreen_lower panel

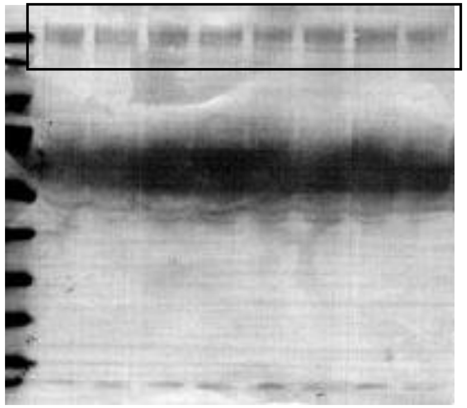

Fig. 4B, α-TIMP-1_left panel

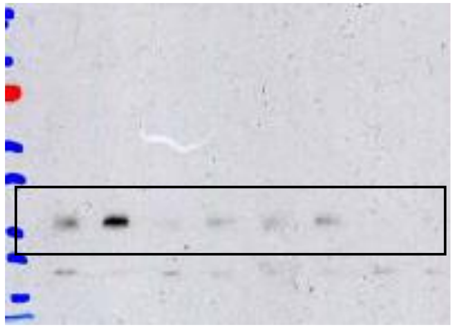

Fig. 4B, FastGreen_left panel

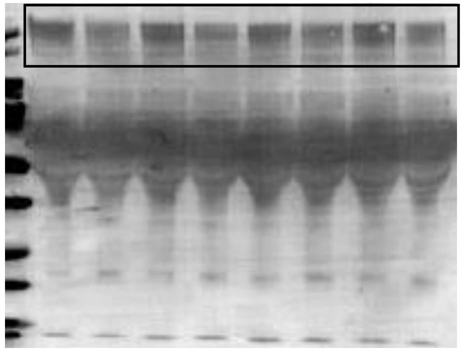

Fig. 4B, α-TIMP-1 right panel

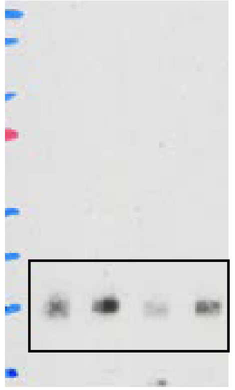

Fig. 4B, FastGreen right panel

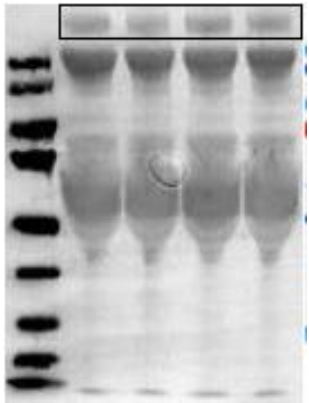

Fig. 4C, α-TIMP-1_6h

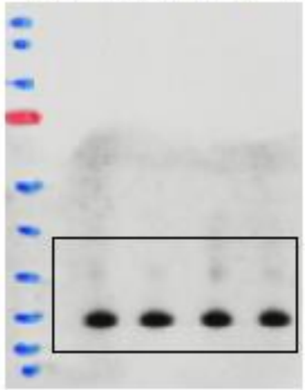

Fig. 4C, α-TIMP_24h

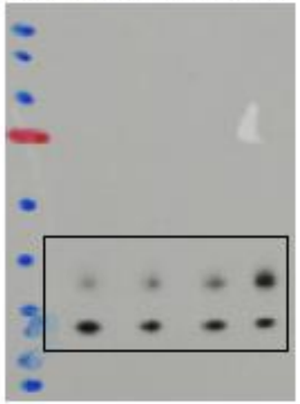

Fig. 4C, FastGreen_6h_Fastgreen_24h

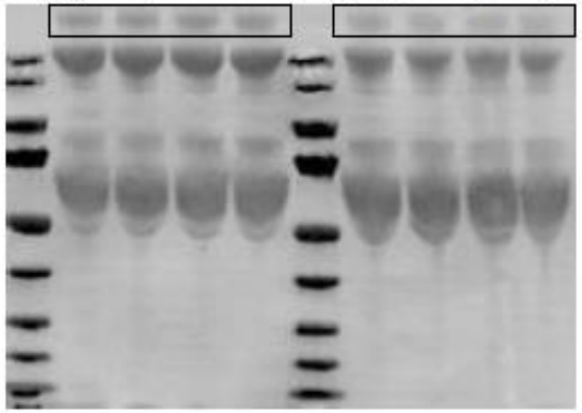

Fig. 4C, α-TiMP-1_right panel

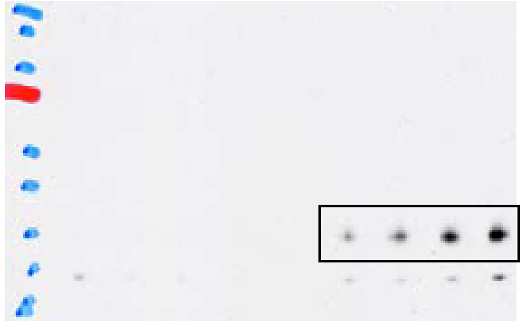

Fig. 4C, FastGreen_right panel

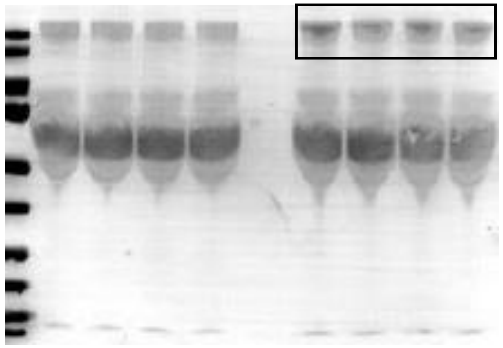

Fig. 6B, α-CD63

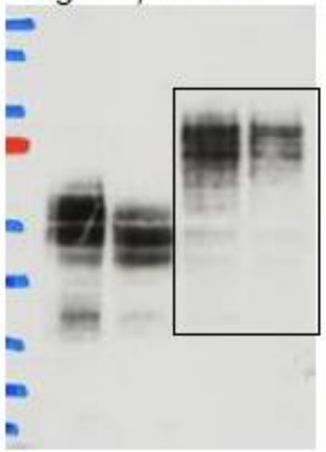

Fig. 6B, FastGreen

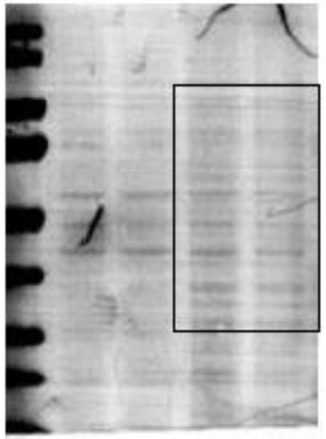

Fig. 6B, α-P-STAT3

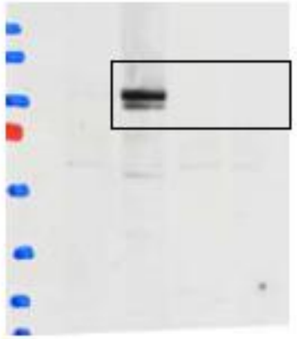

Fig. 6B, α-STAT3

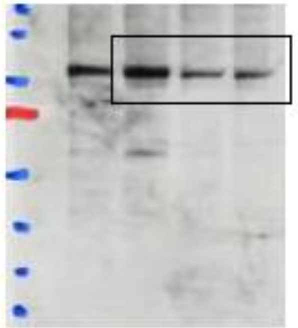

Fig. 6B, α-P-ERK1/2

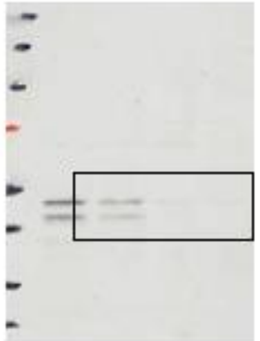

Fig. 6B, α-ERK1/2

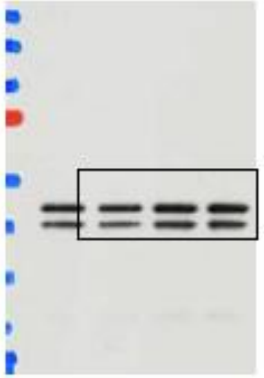

Fig. 6B, FastGreen

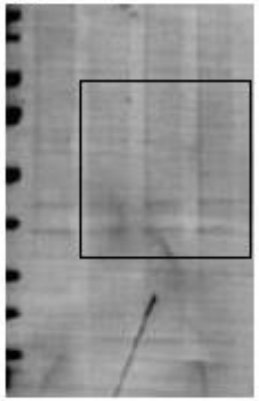

Supplement: Supplementary file 1 [file cancers-14-04983-s001.zip › cancers-1910526-supplementary.pdf]
